# Impacts of high-temperature and humidity transportation on rice quality: an integrated analysis of microbial community succession and flavor compound alterations

**DOI:** 10.3389/fnut.2026.1792369

**Published:** 2026-03-05

**Authors:** Disha Jiang, Yulin Wang, Yun Ling, Sergei A. Eremin, Liliya I. Mukhametova, Jinglin Du, Hao Hu

**Affiliations:** 1 College of Food and Health, Zhejiang Agriculture and Forestry University, Hangzhou, Zhejiang, China; 2 College of Food and Health, Zhejiang Institute of Economics and Trade, Hangzhou, Zhejiang, China; 3 Faculty of Chemistry, M.V. Lomonosov Moscow State University, Moscow, Russia; 4 Zhejiang Provincial Grain and Oil Product Quality Inspection Center, Hangzhou, Zhejiang, China

**Keywords:** high temperature and humidity, metagenomics, microbial community, rice quality, transportation, volatile compounds

## Abstract

This study investigated the dynamic changes in rice quality, microbial communities, and volatile compound profiles during simulated summer transportation (35 °C, 70% RH, 15 days). Indica rice samples were systematically collected every 3 days and analyzed using HS-SPME-GC-MS/MS, HS-GC-IMS, and metagenomic sequencing. Prolonged transportation significantly altered the physicochemical properties of the rice. Moisture content plateaued on day 12, while germination rates declined significantly starting from day 6. Furthermore, fatty acid values increased continuously due to accelerated lipid hydrolysis and oxidation. Visible mold growth became evident on day 12, marking a critical tipping point for quality deterioration. The odor activity value (OAV) and relative odor activity value (ROAV) analyses revealed that the decline in unsaturated fatty aldehydes such as (E)-2-nonenal and the significant accumulation of alcohols, ketones, and short-chain esters, including 1-octen-3-ol and ethyl acetate, drove the transition from a “fresh and fatty” aroma to one characterized by moldy, fermented, and pungent notes. Metagenomic analysis demonstrated a profound ecosystem shift from bacterial dominance (Proteobacteria, Actinobacteria) to fungal dominance. Notably, *Lichtheimia* surged from <0.01% to 23.95%, becoming the dominant genus, while *Aspergillus* increased from 0.03% to 4.57%. Correlation analysis indicated that while *Pseudomonas* was associated with elevated fatty acid levels, the flavor shift was primarily linked to microbial succession. These findings provide insights into the synergistic mechanisms of rice spoilage and suggest that specific volatile markers could serve as early warning indicators for quality control in real-world grain logistics.

## 1 Introduction

Rice (*Oryza sativa* L.) serves as a staple food for over half of the global population, providing essential carbohydrates, proteins, and micronutrients (1). Postharvest quality preservation during storage and transportation is critical for ensuring food security and maintaining nutritional value (2). Postharvest losses of rice can reach 10%–20% in developing countries, with transportation representing one of the most vulnerable stages where quality deterioration occurs. During transportation, rice grains are frequently exposed to fluctuating environmental conditions, particularly elevated temperature and humidity, which accelerate various biochemical and microbiological processes leading to quality degradation (3). Quality deterioration in rice during transportation manifests through multiple interconnected pathways. Physicochemical changes include moisture absorption, which promotes microbial growth; lipid oxidation, which generates off-flavors and rancidity; and enzymatic activities that alter grain composition (4). The germination rate, a key indicator of rice vitality, declines progressively under adverse conditions (5). Concurrently, fatty acid values increase as lipids undergo hydrolysis and oxidation, producing volatile compounds that contribute to undesirable odors. These changes not only compromise sensory attributes but also reduce nutritional quality and market value.

Volatile compounds play a crucial role in determining rice flavor quality and consumer acceptance. The aroma profile of fresh rice is characterized by lipid-derived compounds, particularly unsaturated aldehydes and alcohols with low odor thresholds, which impart pleasant fatty, green, and cereal-like notes (6). During storage and transportation, these desirable compounds degrade through further oxidation and transformation, while deterioration-related metabolites accumulate. Advanced analytical techniques including gas chromatography-mass spectrometry (GC-MS) and gas chromatography-ion mobility spectrometry (GC-IMS) have enabled comprehensive profiling of volatile compounds, providing insights into flavor formation and deterioration mechanisms (7, 8). The rice grain ecosystem harbors a diverse microbial community comprising bacteria, yeasts, and molds that interact dynamically with the substrate and environmental conditions (9). Under favorable conditions, beneficial microorganisms may predominate; however, elevated temperature and humidity create selective pressures favoring spoilage organisms, particularly filamentous fungi (10). These microorganisms contribute to quality deterioration through multiple mechanisms: direct tissue invasion, production of hydrolytic enzymes (lipases, proteases, amylases), and generation of secondary metabolites including off-flavor compounds (11). Metagenomic sequencing has revolutionized our understanding of complex microbial communities, enabling taxonomic characterization and functional prediction of rice-associated microbiota (12). Despite growing recognition of the importance of transportation conditions in rice quality maintenance, comprehensive studies integrating volatile compound dynamics with microbial community succession under controlled high-temperature and high-humidity conditions remain limited (13). Previous research has primarily focused on either physicochemical changes or microbial contamination separately, lacking an integrated perspective on the interconnections between environmental stress, microbial responses, and flavor compound alterations (14). The specific mechanisms by which microbial succession drives volatile compound transformations during transportation are not fully understood.

This study addresses these knowledge gaps by employing an integrated analytical approach to investigate the impacts of simulated summer transportation conditions on rice quality. Specifically, we aimed to: (1) characterize the dynamic changes in physicochemical properties including moisture content, germination rate, and fatty acid values during transportation; (2) profile volatile compound alterations using complementary GC-MS/MS and GC-IMS techniques; (3) elucidate microbial community succession patterns through metagenomic sequencing; and (4) establish correlations between microbial genera, physicochemical indicators, and key volatile compounds. By integrating multi-omics data, this research provides novel insights into the microbiological mechanisms underlying rice quality and flavor changes during transportation, offering a scientific basis for developing effective quality control and risk management strategies.

## 2 Materials and methods

### 2.1 Chemical reagents

Anhydrous ethanol and potassium hydroxide were procured from Sinopharm Chemical Reagent Co., Ltd. Chromatographic-grade 2-octanol was acquired from Shanghai Yuanye Bio-Technology Co., Ltd. Other analytical-grade reagents, such as 2-butanone, 2-pentanone, 2-hexanone, 2-heptanone, 2-octanone, and 2-nonanone, were purchased from Shanghai Aladdin Biochemical Technology Co., Ltd.

### 2.2 Preparation of rice grain samples

All indica rice samples used in this study were obtained from the same batch of paddy rice collected from the Shaoxing Grain Depot of Zhejiang Provincial Grain Reserve Group Co., Ltd. A portion of the rice was analyzed immediately at the start of the experiment (Day 0) to serve as the control group, representing the baseline quality prior to simulated transportation. A constant temperature and humidity incubator (LHS-80HC-II, Shanghai Yiheng Scientific Instrument Co., Ltd.) was used to simulate summer transportation conditions. The initial temperature and relative humidity (RH) were set at 35 °C and 70%, respectively, and the simulation period lasted for 15 days. To replicate the dynamic environmental fluctuations typical of real-world transportation, the temperature and RH within the incubator were increased by 2.5 °C and 2.5% every 3 days immediately following sampling. Subsequent to sampling. Collected samples were promptly frozen in liquid nitrogen and stored at −80 °C for further analyses.

### 2.3 Analysis of physicochemical properties

The germination rate was determined according to the method specified in the National Standard of China GB 5520-2011. The moisture content was measured using the direct drying method as per GB 5009.3-2016. The fatty acid value was determined via manual titration, following the procedure described in GB 5510-2024.

### 2.4 Analysis of volatile compounds

#### 2.4.1 Determination of volatile compounds in rice grains by HS-SPME-GC-MS/MS

The analysis was adapted from the method reported by Qiu et al. (9) with minor modifications. Briefly, 4.0 g of ground rice sample was accurately weighed into a 20 mL headspace vial. Subsequently, 100 μL of internal standard solution (2-octanol, 0.05 mg/mL) was added. The sample was equilibrated at 60 °C for 40 min, and then the analytes were desorbed at 250 °C for 3 min in the GC injector. Volatile compounds were analyzed using a GC-MS/MS system (Agilent 8890-7000E, Agilent Technologies, USA) equipped with an HP-5MS capillary column (30 m × 0.32 mm × 0.25 μm, Agilent Technologies, USA). High-purity helium (>99.99%) was employed as the carrier gas at a constant flow rate of 1.0 mL/min in splitless mode.

The column temperature program was set as follows: initial temperature held at 50 °C for 2 min, increased to 125 °C at a rate of 8 °C/min and held for 3 min, raised to 165 °C at 4 °C/min and held for 2 min, and finally ramped to 230 °C at 10 °C/min and held for 2 min. The mass spectrometer was operated in electron impact (EI) mode at 70 eV, with the ion source transfer line temperatures set at 230 °C and 250 °C, respectively. Mass spectra were acquired across the range of *m/z* 50–550. Volatile compounds were identified by comparing mass spectra with the NIST 20 library and linear retention indices (LRI), and semi-quantitative analysis was conducted using the internal standard method.

#### 2.4.2 Determination of volatile compounds in rice grains by HS-GC-IMS

The analysis was adapted from the method reported by Xiong et al. (15) with minor modifications. Volatile compounds were analyzed via a gas chromatography-ion mobility spectrometry system (GC-IMS; FlavourSpec^®^, G.A.S., Germany). Briefly, 2.0 g of the rice grain sample was accurately weighed into a 20 mL headspace vial. The sample was incubated at 60 °C for 15 min with agitation at 500 rpm. Subsequently, 500 μL of the headspace gas was automatically injected in splitless mode using a headspace syringe maintained at 85 °C. Separation was carried out on a gas chromatograph equipped with an MXT-5 capillary column (15 m × 0.53 mm × 1.0 μm). The column temperature was maintained at 60 °C, and the total analysis time was 20 min. High-purity nitrogen was used as the carrier gas with the flowing flow program: 2 mL/min (0–2 min), increased to 10 mL/min from (2–10 min), and finally to 100 mL/min from (10–20 min). After chromatographic separation, analytes were introduced into the ion mobility spectrometer (IMS) for detection. Ionization was performed using a tritium (^3^H) source in positive ion mode. The drift tube was maintained at 45 °C using nitrogen drift gas (>99.999%) at a flow rate of 150 mL/min. A homologous series of C4-C9 ketones (2-butanone, 2-pentanone, 2-hexanone, 2-heptanone, 2-octanone, and 2-nonanone) was utilized as external reference to calculate the retention indices (RI) for qualitative identification.

#### 2.4.3 Evaluation of volatile compounds

The influence of high-temperature and high-humidity transportation on rice flavor was assessed through the calculation of the odor activity values (OAVs) and relative odor activity values (ROAVs) of volatile organic compounds detected by HS-SPME-GC-MS/MS and HS-GC-IMS. The odor activity value (OAV) was defined as the ratio of the concentration of an aroma compound to its odor threshold. Compounds having OAVs greater than 1 were regarded as making a substantial contribution to the overall aroma profile of the sample (16). OAVs were calculated according to Equation 1.


$$O\,A\,V=\frac{C\,i}{O\,T\,i}$$

where (Ci) is the concentration of volatile compound i (μg/kg), and (OTi) is the odor threshold of this compound in water (μg/kg).

The relative odor activity value (ROAV) is a quantitative index used to evaluate the relative contribution of individual volatile compounds to the overall flavor of a complex mixture. Compounds with ROAV values ≥ 1 are considered the main contributors to the overall flavor of the sample (17). ROAVs were calculated according to Equation 2.


$$R\,O\,A\,V\,i\approx 100\times \frac{A\,i}{A\,s}\times \frac{T\,s}{T\,i}$$

where (*Ai*) is the peak area of volatile compound *i*; (*Ti*) is the sensory threshold of volatile compound *i* (μg/L); (*As*) is the peak area of the compound that contributes most to the overall flavor of the sample; and (*Ts*) is the sensory threshold of the compound that contributes most to the overall flavor of the sample (μg/L).

### 2.5 Metagenomic sequencing and analysis

Total genomic DNA was extracted from rice grain samples using the Fecal Genome DNA Extraction Kit (AU46111-96, BioTeke, China), following the manufacturer’s instructions. DNA libraries were constructed using the TruSeq Nano DNA Library Preparation Kit-Set (FC-121-4001, Illumina, USA). Subsequently, the metagenomic libraries were sequenced on an Illumina NovaSeq 6000 platform with paired-end 150 bp reads (PE150) at LC-Bio Technology Co., Ltd., (Hangzhou, China). Raw sequencing reads were filtered using fastp (v0.23.4) to remove adaptor contamination, low-quality reads, and reads containing undetermined bases. High-quality reads were aligned to the rice reference genome using Bowtie (v2.2) to remove host sequences. The remaining clean reads were assembled *de novo* using MEGAHIT (v1.2.9) and used for downstream microbial taxonomic and functional analyses.

MetaGeneMark (v3.26) was applied to predict coding sequences (CDSs) from the assembled contigs. All predicted CDSs were clustered by means of MMseq2 (v15-6f452) to generate a non-redundant gene catalog (unigenes). Taxonomic annotation of the rice-associated microbiota was carried out using DIAMOND (v0.9.14) against the NR database. Differentially abundant microbial species were identified through the application of the Wilcoxon rank-sum test, with statistical significance defined as *P* < 0.05 and | log2 fold change| > 1. Functional annotation of microbial genes was performed based on the Kyoto Encyclopedia of Genes and Genomes (KEGG) database.

### 2.6 Statistical analysis

All experiments were performed with a minimum of three independent replicates, and the outcomes are presented as the mean ± standard deviation (SD). Statistical analyses were executed using SPSS 27.0 software (SPSS Inc., Chicago, USA). Disparities among groups were assessed via one - way analysis of variance (ANOVA), and a *P*-value < 0.05 was regarded as statistically significant. Figures were produced using Origin 2021 (OriginLab, Northampton, USA) and SIMCA 14.1 (Umetrics, Umeå, Sweden). Hierarchical clustering heatmaps of rice volatile compounds and correlation bubble heatmaps between microbial genera and physicochemical indicators were generated by means of the Metware Cloud Platform.^1^ Correlation networks between microbial genera and volatile compounds were constructed utilizing Cytoscape 3.10.4 software.

## 3 Results and discussion

### 3.1 Changes in physicochemical properties of rice grains

Transportation is a critical stage affecting the postharvest quality of rice grains, as temperature and humidity fluctuations can progressively alter the physiological status and internal composition of rice, leading to quality deterioration (18). Moisture content, germination rate, and fatty acid value are key physicochemical indicators for evaluating quality stability and deterioration during storage and transportation (19), and their dynamic changes effectively reflect the impact of transportation conditions on rice quality.

**FIGURE 1:**
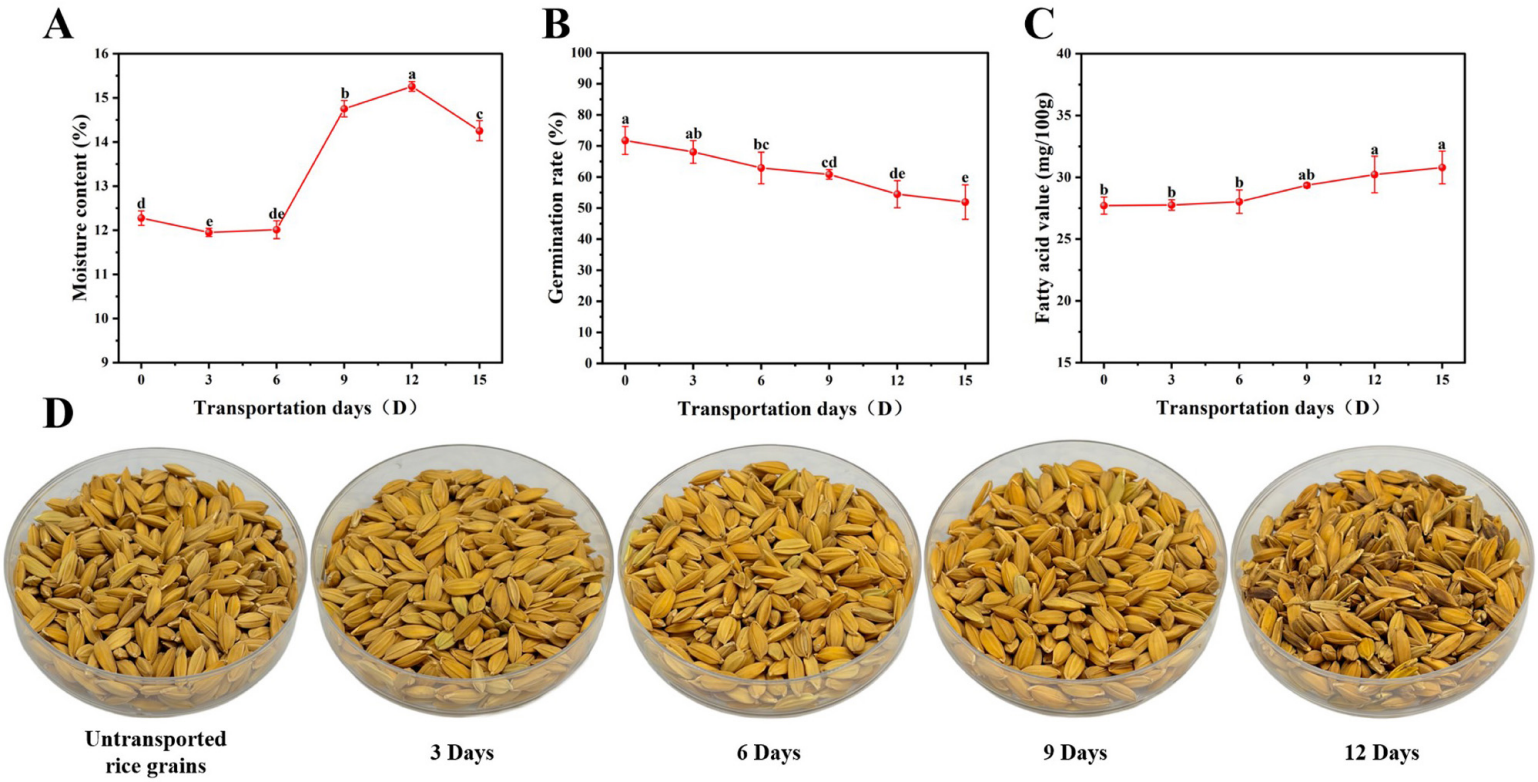
Changes in physicochemical properties and appearance of rice grains during transportation. **(A)** Moisture content; **(B)** germination rate; **(C)** fatty acid value; **(D)** appearance changes of rice grains during transportation at 0, 3, 6, 9, and 12 days. The letters a-e indicate significant differences (*p* < 0.05): identical letters represent no significant difference, while different letters indicate significant differences.

During the simulated transportation period, moisture content remained relatively stable in the early stage before gradually increasing to a peak on day 12 (Figure 1A). This was followed by a slight decrease on day 15; however, the overall level remained significantly higher than the initial value. This subsequent slight decline could be attributed to the loss of grain integrity. Specifically, fungal invasion compromised the seed coat and cell wall structures during the late stage of transportation, thereby altering their hygroscopic properties. Concurrently, the local moisture equilibrium may have been influenced by metabolic heat generated by vigorous microbial activity. Simultaneously, the germination rate exhibited a continuous downward trend (Figure 1B), showing a significant decrease from day 6 onward and reaching its lowest level between days 12 and 15. This indicates that high temperature and high humidity conditions markedly inhibited the physiological activity of the rice grains. The fatty acid value increased steadily, with a more pronounced rise in the later stages (Figure 1C). Values on days 12 and 15 were significantly higher than the initial level, reflecting the continuous lipid hydrolysis and oxidation under high temperature and high humidity conditions.

In addition to physicochemical changes, alterations in rice appearance provided direct visual evidence of quality deterioration (Figure 1D). The untransported rice grains were plump, uniform in color, and golden, with dry and intact husks. By day 3 of transportation, the grains exhibited only a slight loss of luster. By day 6, slight browning emerged at the grain edges. By day 9, the rice appeared noticeably darker, with mottled yellow-brown patches, and some grains showed slight shrinkage and sporadic mold spots. By day 12, mold had spread across the grains, which were extensively browned, shriveled, and lacked gloss, indicating pronounced fungal growth. At this stage, rice grains exhibited high moisture content, markedly reduced germination rate, and continuously increasing fatty acid values. These observations indicate that rice quality had transitioned from an early deterioration stage dominated by physicochemical changes to a pronounced deterioration stage driven by microbial growth. Therefore, rice samples collected on day 12 were selected as the representative treatment group for subsequent analyses.

### 3.2 Changes in volatile compounds

#### 3.2.1 GC–MS/MS-based analysis of volatile compounds in rice grains

**FIGURE 2:**
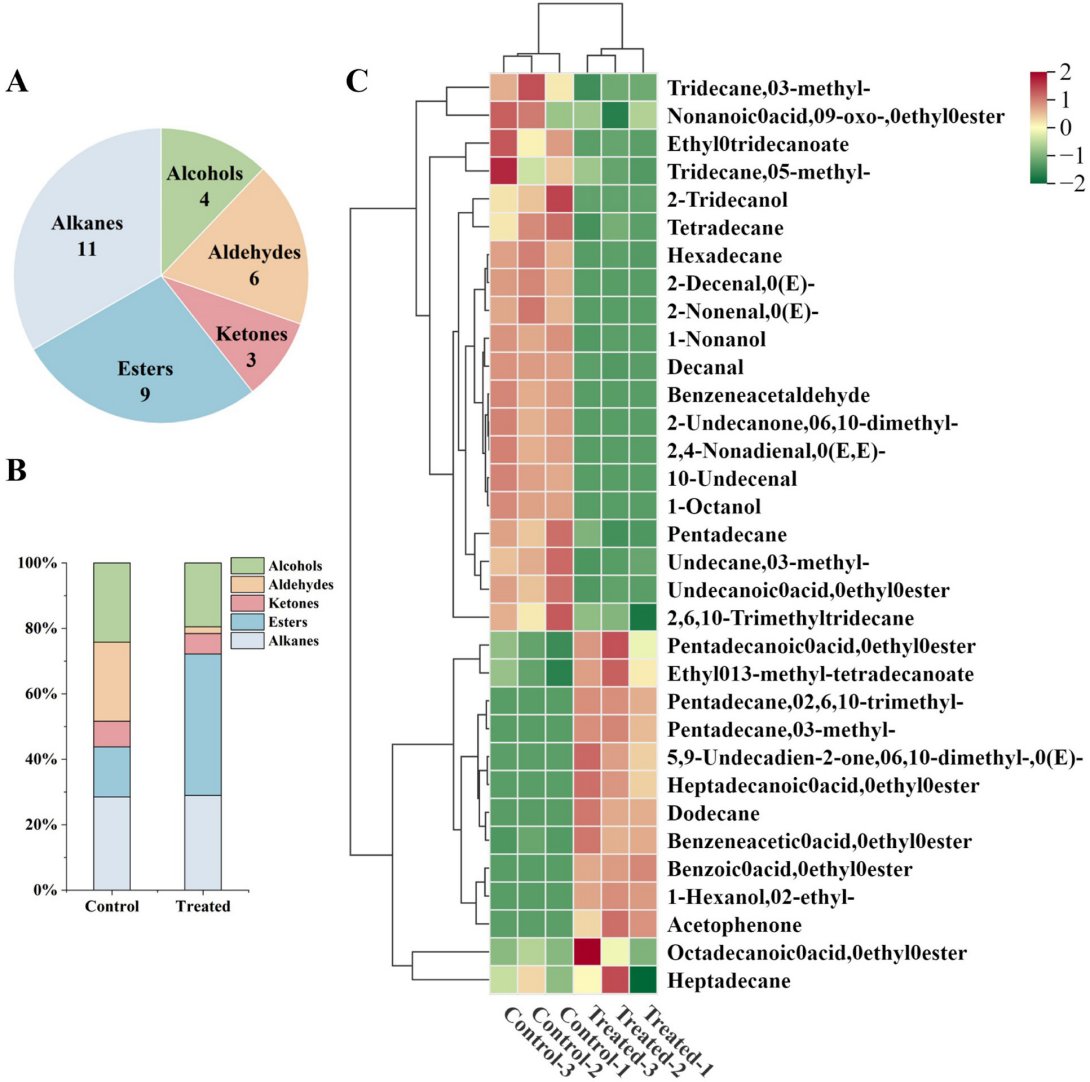
Gas chromatography-mass spectrometry/mass spectrometry (GC-MS/MS) analysis of volatile compounds in rice grains after transportation. **(A)** Category distribution of volatile compounds; **(B)** relative content distribution of volatile compounds between groups; **(C)** hierarchical clustering heatmap of volatile compounds.

In this study, GC-MS/MS was employed to analyze the dynamic changes in the volatile compounds of rice after 12 days of simulated transportation. A total of 33 volatile compounds were identified (Supplementary Table 1), encompassing four alcohols, six aldehydes, three ketones, nine esters, and eleven alkanes (Figure 2A). Simulated high-temperature and high-humidity transportation significantly altered the volatile profile of the rice (Figure 2B), with changes primarily concentrated in aldehydes and esters. The heatmap analysis (Figure 2C) visually illustrated the transition from “fresh” to “deteriorated” flavor profiles. The control group was dominated by aldehydes and certain medium-to long-chain alcohols, specifically characterized by high levels of unsaturated aldehydes such as (E)-2-decenal, (E)-2-nonenal, (E,E)-2,4-nonadienal, decanal, and benzeneacetaldehyde. As typical products of mild lipid oxidation, these compounds imparted fresh fatty and grassy notes to the rice (20).

**FIGURE 3:**
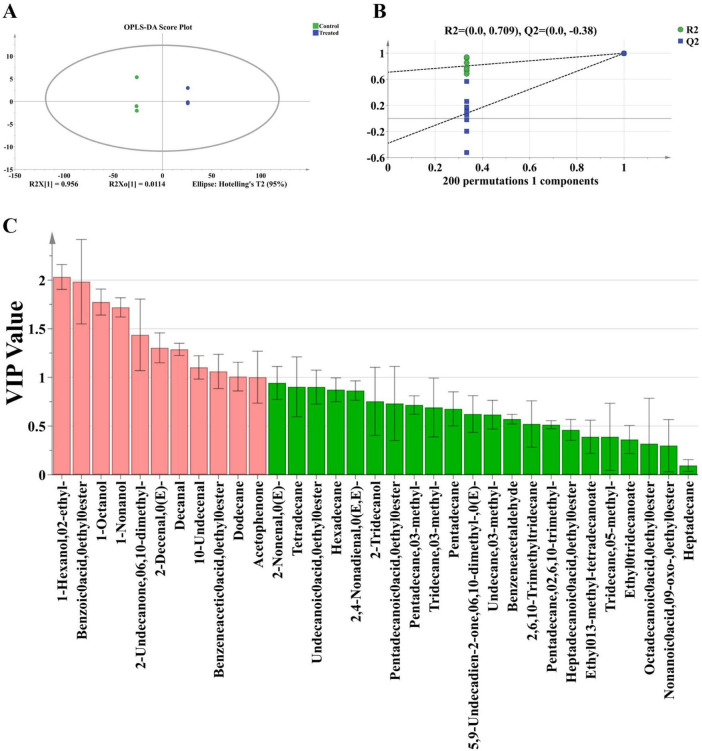
OPLS-DA model analysis of volatile compounds discriminating between control and treated rice samples. **(A)** OPLS-DA score plot; **(B)** permutation test of the OPLS-DA model; **(C)** VIP scores (VIP ≥ 1) of key volatile compounds.

However, in the treated group, the aforementioned aldehydes were either undetectable or significantly reduced, with only trace amounts of decanal remaining. This indicates that the simulated transport conditions accelerated the secondary degradation of unsaturated fatty acid oxidation products (21), which likely contributed to the loss of fresh aroma. Regarding alcohols, 1-octanol, 2-tridecanol, and 1-nonanol detected in the control group were significantly reduced or completely absent in the treated group, whereas 2-ethyl-1-hexanol newly emerged after transportation. This shift implies a transition in alcohol composition from lipid-derived background components to compounds related to deterioration or metabolism, as 2-ethyl-1-hexanol is commonly associated with the oxidative deterioration of grains and potential microbial metabolic processes (22). Ester compounds generally displayed an increasing trend in the treated group, which is typically ascribed to the enhanced fatty acid degradation and esterification reactions under high temperature and high humidity conditions (23). In contrast, alkanes exhibited relatively minor changes and mainly represent background products of lipid degradation, contributing minimally to the overall aroma. Their relative stability further indicates that flavor changes in rice grains were primarily concentrated in oxygenated volatile compounds (24). To characterize the differences in volatile profiles after transportation, OPLS-DA was conducted. The score plot (Figure 3A) demonstrated that all samples were situated within the 95% confidence interval, and a distinct separation was observed between the transported rice samples and the control group in terms of volatile compound composition. Permutation testing further confirmed the robustness and stability of the model (Figure 3B), with no evidence of underfitting or overfitting.

The contribution of individual volatile compounds to the differences between sample groups was evaluated using variable importance in projection (VIP) values, with VIP ≥ 1 commonly employed as the criterion for identifying differential compounds (25). By integrating the VIP values from the OPLS-DA model (VIP ≥ 1) with the results of significance testing (*P* < 0.05), a total of 11 volatile compounds exhibiting significant differences after transportation were identified (Figure 3C). These compounds can be regarded as key characteristic markers associated with the transportation process and could potentially serve as early warning indicators for rice quality deterioration.

#### 3.2.2 GC–IMS-based analysis of volatile compounds in rice grains

**FIGURE 4:**
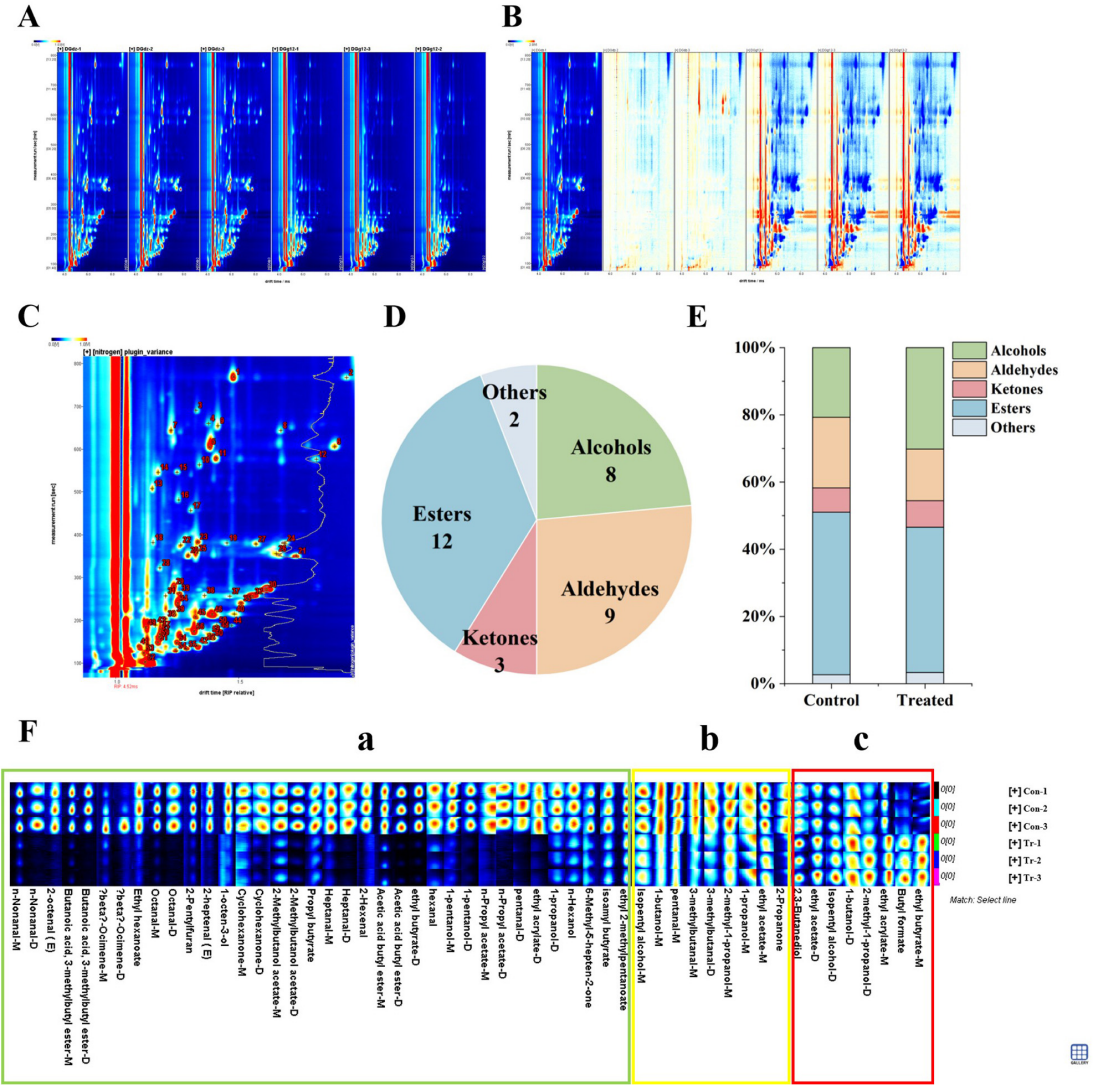
Gas chromatography-ion mobility spectrometry (GC-IMS) analysis of volatile compounds in rice grains after transportation. **(A)** Two-dimensional GC-IMS spectrum; **(B)** two-dimensional difference spectrum; **(C)** qualitative characterization of volatile compounds based on GC-IMS; **(D)** category distribution of volatile compounds; **(E)** relative content distribution of volatile compounds between groups; **(F)** fingerprint profile of volatile compounds. The a-c is substances that are used to distinguish different behavioral characteristics based on the changing patterns of volatile compounds.

To complement the GC-MS/MS analysis and provide a rapid, visual overview of volatile profiles, headspace-gas chromatography-ion mobility spectrometry (HS-GC-IMS) was employed. The results revealed substantial differences in volatile compound signals between the control and treated groups across both retention and drift time dimensions (Figure 4A). Using one control sample (Control-1) as a reference (Figure 4B), spectra from other controls showed high similarity, whereas treated samples exhibited distinct deviations, indicating that transportation conditions significantly altered the volatile profile.

Headspace-gas chromatography-ion mobility spectrometry detected a total of 62 volatile signals across all samples (Figure 4C). Among them, 34 volatile compounds (including monomers and dimers) were identified, comprising eight alcohols, nine aldehydes, three ketones, twelve esters, and two other types of compounds (Figure 4D). The relative contents of these compounds are presented in Supplementary Table 2. Following the 12-days transportation, the volatile composition was markedly reconfigured (Figure 4E), Overall, these compositional shifts were consistent with a transition in the sensory profile from a fresh, lipid-derived aroma to one characterized by odors related to deterioration and metabolism, In the treated group, the overall proportion of alcohols increased. Small-molecule alcohols, such as 1-octen-3-ol and 2,3-butanediol, showed significant rises, while 1-pentanol decreased. This suggests that alcohols associated with oxidative reactions and potential metabolic processes gradually became predominant during transportation. Aldehydes, which were the most abundant class in the control group and are closely related to the fresh aroma of rice (26), exhibited a pronounced overall decline in the treated group. In particular, straight-chain or unsaturated aldehydes with low odor thresholds, such as nonanal, octanal, heptanal, (E)-2-octenal, and hexanal, decreased significantly, while only 3-methylbutanal and pentanal showed moderate increases. These changes indicate that the primary lipid oxidation products were continuously consumed during transportation, accompanied by the gradual accumulation of branched-chain aldehydes. In the treated group, the relative content of ketones increased overall, with remarkable increases in 6-methyl-5-hepten-2-one and cyclohexanone, suggesting further advancement of lipid oxidation. In contrast, the changes in ester compounds were more intricate. Esters with relatively high proportions in the control group, like butyl acetate and ethyl butyrate, decreased significantly in the treated group, while small-molecule esters including ethyl acetate, ethyl acrylate, isoamyl butyrate, and propyl butyrate increased notably. This led to a shift in the ester-related aroma from predominantly fruity and sweet notes to more pungent and fermentation-like characteristics.

Based on the compositional characteristics of volatile compounds in the two groups, a volatile compound fingerprint was constructed (Figure 4F). In this fingerprint, the signal intensity is represented by color intensity, where brighter colors signify stronger signals (27). Pronounced differences between the control and treated samples were mainly concentrated in regions a and c, while most compounds in region b remained relatively stable. In region a, several well-known volatile compounds, such as nonanal, (E)-2-octenal, 3-methylbutyl butyrate, β-ocimene, ethyl hexanoate, octanal, and 2-pentylfuran, decreased significantly after transportation. Conversely, volatile compounds in region c gradually increased, including 2-methyl-1-propanol-Jinglin D, ethyl acrylate-M, butyl formate, and ethyl butyrate-M. The differential abundance of these compounds could serve as potential indicators for rice quality deterioration during transportation.

#### 3.2.3 Analysis of key volatile compounds

According to the odor activity value (OAV) analysis based on GC-MS/MS (Supplementary Table 1), high OAVs were observed for various unsaturated and straight-chain fatty aldehydes in the rice prior to transportation. Among these, compounds such as (E)-2-nonenal, (E, E)-2,4-nonadienal, (E)-2-decenal, and decanal were identified as the primary contributors to the fresh fatty and grain-like aroma. Following transportation, most of these aforementioned key aldehydes were not detected, or their OAV values were found to be significantly reduced. It is suggested that under sustained high-temperature and high-humidity conditions, the aroma-active intermediates in lipid oxidation pathways were further decomposed or transformed. Concurrently, an accumulation of specific alcohols was observed, which imparted faint fermented and rancid characteristics were presented, marking the transition from a fresh to a deteriorated aroma profile. A relatively low overall contribution at the OAV level was exhibited by alcohols, esters, and alkanes, which were characterized primarily as background odor components during the quality deterioration process.

Furthermore, the relative odor activity value (ROAV) analysis based on gas chromatography - ion mobility spectrometry (GC-IMS) clarified the dominant alterations in the aroma structure after transportation (Supplementary Table 2). Although the relative content of typical fatty aldehydes decreased, significantly elevated ROAVs were recorded for 3-methylbutanal, pentanal, and certain unsaturated aldehydes. Due to their extremely low odor thresholds, these compounds contributed to pronounced pungent and aging odor characteristics. A marked increase was observed in the ROAVs of alcohols, including 1-octen-3-ol, isopentyl alcohol, 2-methyl-1-propanol, 1-butanol, and 1-propanol. These changes were attributing to enhanced microbial metabolism and amino acid degradation, contributing to prominent fermented and moldy notes (28). Additionally, the ROAV profile of esters shifted from fruity and sweet contributors such as acetic acid butyl ester and ethyl butyrate toward more pungent compounds, including ethyl acrylate and other short-chain esters (29, 30), thereby further exacerbating the development of undesirable odors in rice grains. Based on the dynamic changes in concentration and the analysis of key volatiles, seven compounds, including 3-methylbutanal, pentanal, 1-octen-3-ol, isopentyl alcohol, 2-methyl-1-propanol, ethyl acrylate, and ethyl acetate-were identified as the characteristic volatile markers indicative of rice deterioration. Crucially, these specific volatiles could serve as potential early indicators for monitoring rice flavor and quality loss, thereby offering significant practical value for grain storage and transportation management.

### 3.3 Metagenomic analysis of microbial communities

**FIGURE 5:**
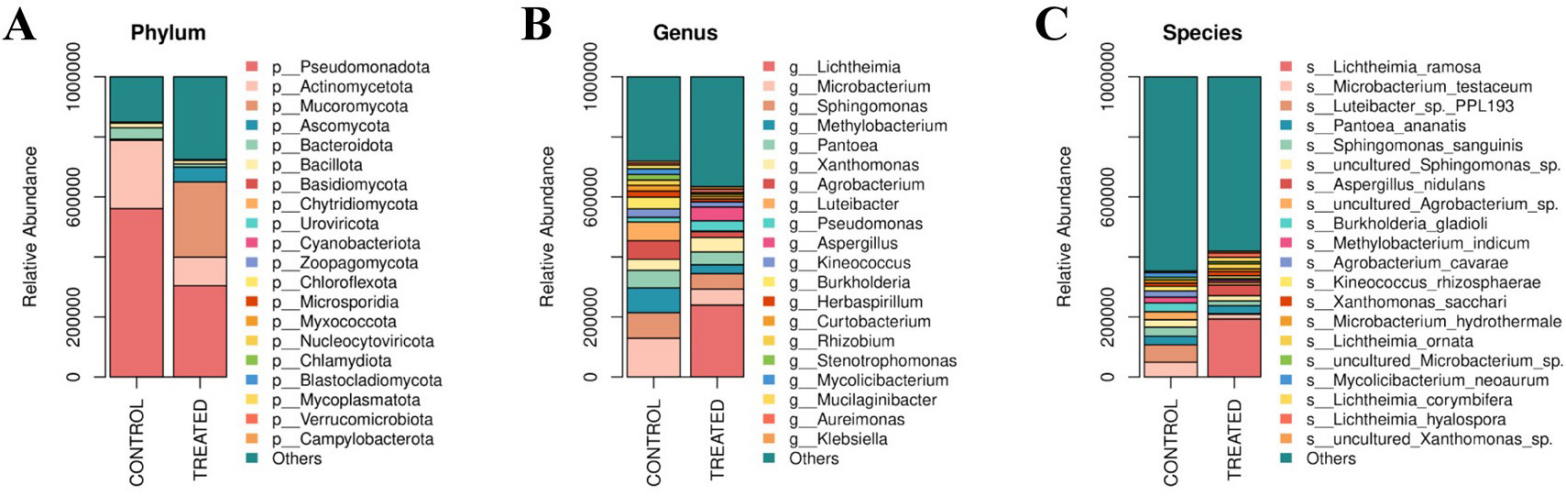
Composition and functional prediction of microbial communities in rice grains during transportation. **(A)** Microbial community composition at the phylum level; **(B)** microbial community composition at the genus level; **(C)** microbial community composition at the species level.

During the rice transportation process, a total of 87 phyla, 191 classes, 375 orders, 799 families, 2,072 genera, and 10,481 species were identified. At the phylum level (Figure 5A), the control group was dominated by Proteobacteria and Actinobacteria. In the treated group, a decline in the abundance of both phyla was observed, whereas the abundance of Mucoromycota was substantially increased to 25.02%, establishing it as one of the major fungal phyla. At the genus level (Figure 5B), the microbial community in the control group was characterized by bacterial dominance, with *Microbacterium* exhibiting the highest abundance, followed by *Sphingomonas* and *Methylobacterium*. However, the treated group showed a widespread decline in the originally dominant bacteria, accompanied by a surge in the fungal genus *Lichtheimia*. Given its unique thermophilic properties, *Lichtheima* gained a competitive advantage under the 35 °C transportation condition, enabling it to proliferate rapidly and outcompete mesophilic fungi. Consequently, its abundance surged from a negligible <0.01% to 23.95%. A significant increase was also recorded for another fungal genus, *Aspergillus*, which rose from 0.03% to 4.57%. At the species level (Figure 5C), bacteria such as *Microbacterium testaceum* in the control group were replaced by *Lichtheimia ramosa* in the treatment group, which was identified as the absolute dominant species (accounting for nearly 20%). Significant increases were also detected in the abundance of other mold-associated fungi, such as *Lichtheimia ornata*, *Lichtheimia corymbifera*, and *Aspergillus nidulans*. Overall, a significant succession of the microbial community from a “bacterial-dominated” to a “fungal-dominated” mold-associated profile was driven by the high-temperature and high-humidity conditions (9). A distinct “inverse” pattern was exhibited, characterized by the explosive proliferation of *Lichtheimia* and *Aspergillus* contrasted against the marked subsidence of bacteria such as *Microbacterium*. These results strongly suggested that during the later stages of stress, rapidly proliferating filamentous fungi exerted strong competitive inhibition or antagonistic effects on the indigenous bacterial community (31). These effects were considered to be the primary drivers of the fundamental restructuring of the community, potentially achieved through the occupation of living space, competition for resources such as carbohydrates, or the secretion of antimicrobial metabolites.

**FIGURE 6:**
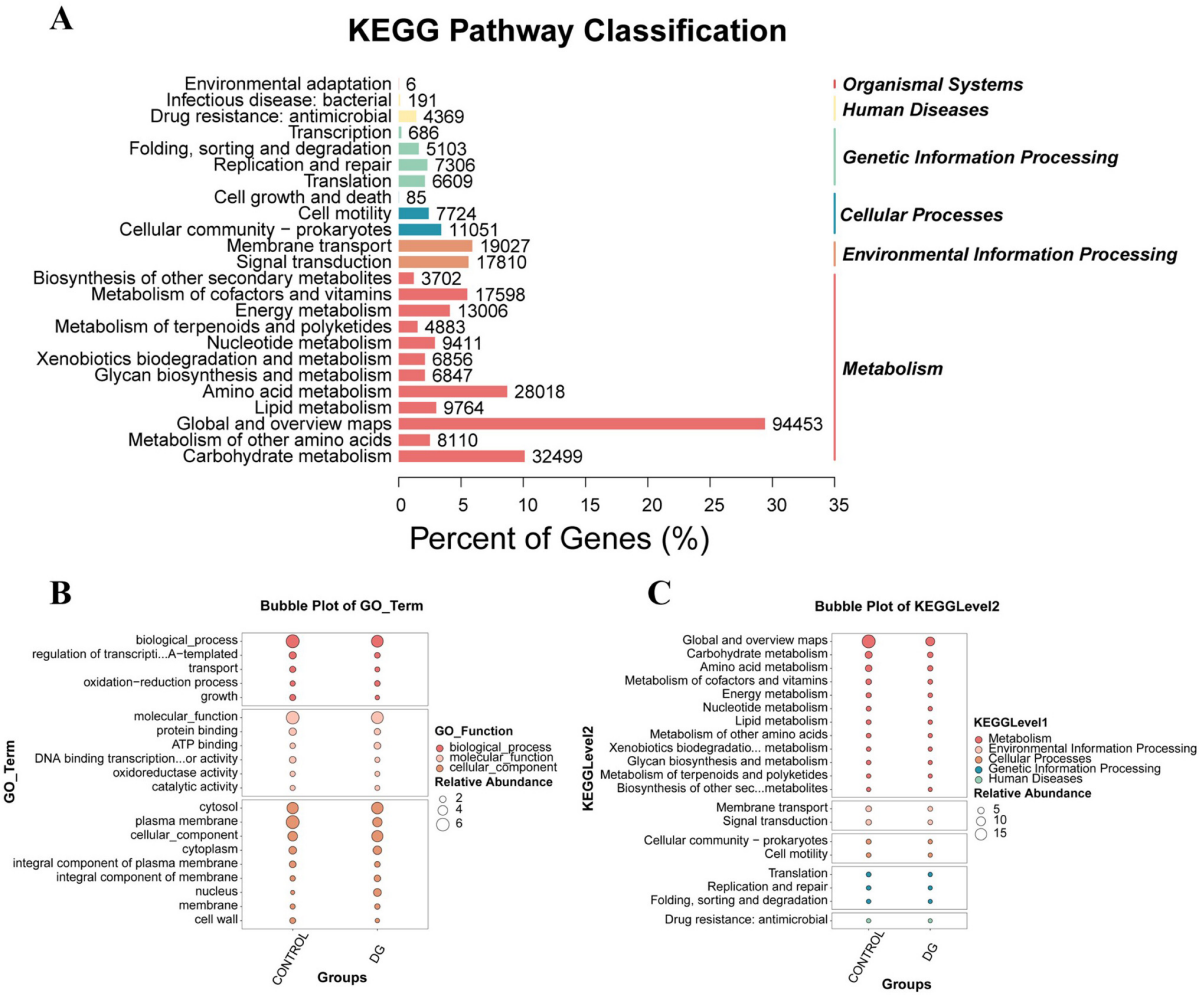
Functional annotation and pathway analysis of microbial communities. **(A)** KEGG level 1 functional classification; **(B)** bubble plot of KEGG level 2 functional pathways; **(C)** Gene Ontology functional annotation of microbial communities, including biological process, molecular function, and cellular component.

To elucidate the functional changes underlying community succession, KEGG pathway annotation was performed. At level 1 annotation (Figure 6A), metabolic functions accounted for the highest proportion, indicating that the metabolic activities of both microorganisms and the rice grains themselves were dominant during the transportation process. Level 2 pathway analysis (Figure 6B) further revealed that the upregulation of specific metabolic pathways was significantly induced by the high-temperature and high-humidity treatment; notably, pronounced changes were observed in lipid metabolism and carbohydrate metabolism pathways. Specifically, the significant enrichment of genes related to lipid metabolism in the treatment group was found to be highly consistent with the continuous elevation of fatty acid values, suggesting that lipid hydrolysis and oxidation may have been accelerated by lipases secreted by microorganisms (particularly molds). Meanwhile, the active pattern of carbohydrate metabolism pathways was corroborated by the exploitative consumption of stored starch during the rapid proliferation of fungi (32). Furthermore, the enrichment of pathways associated with environmental adaptation, such as “Antimicrobial resistance” and “Signal transduction,” provided further evidence that complex interspecies interactions and competition were involved in the process of community restructuring.

Gene Ontology functional annotation (Figure 6C) further revealed significant shifts in the gene expression profile. In terms of biological processes, an upregulation of genes associated with oxidation-reduction processes was observed, suggesting that an oxidative stress response might have been triggered by the stress conditions. Simultaneously, transcriptional regulation genes were active, whereas growth-related genes were downregulated. Regarding molecular functions, genes related to oxidoreductase activity and catalytic activity were notably upregulated. For cellular components, the downregulation of plasma membrane-related genes and the upregulation of nucleus-related genes implied that membrane stability might be compromised, suggesting the concurrent activation of adaptive responses such as intranuclear DNA repair (33). Collectively, these functional gene alterations correspond well with the phenotypic deterioration of rice quality. The upregulation of redox-process-related genes is consistent with the observed rise in fatty acid values and lipid oxidation. The active carbohydrate and amino acid metabolic pathways likely reflect the consumption of nutrients during fungal proliferation and the subsequent decline in germination rate. Furthermore, the downregulation of cell membrane genes and the upregulation of stress response genes offer a cellular-level explanation for the loss of grain viability. These associations provide further support for the link between microbial gene expression changes and the phenotypic decay of rice.

### 3.4 Correlation analysis between microbial genera and physicochemical indicators

**FIGURE 7:**
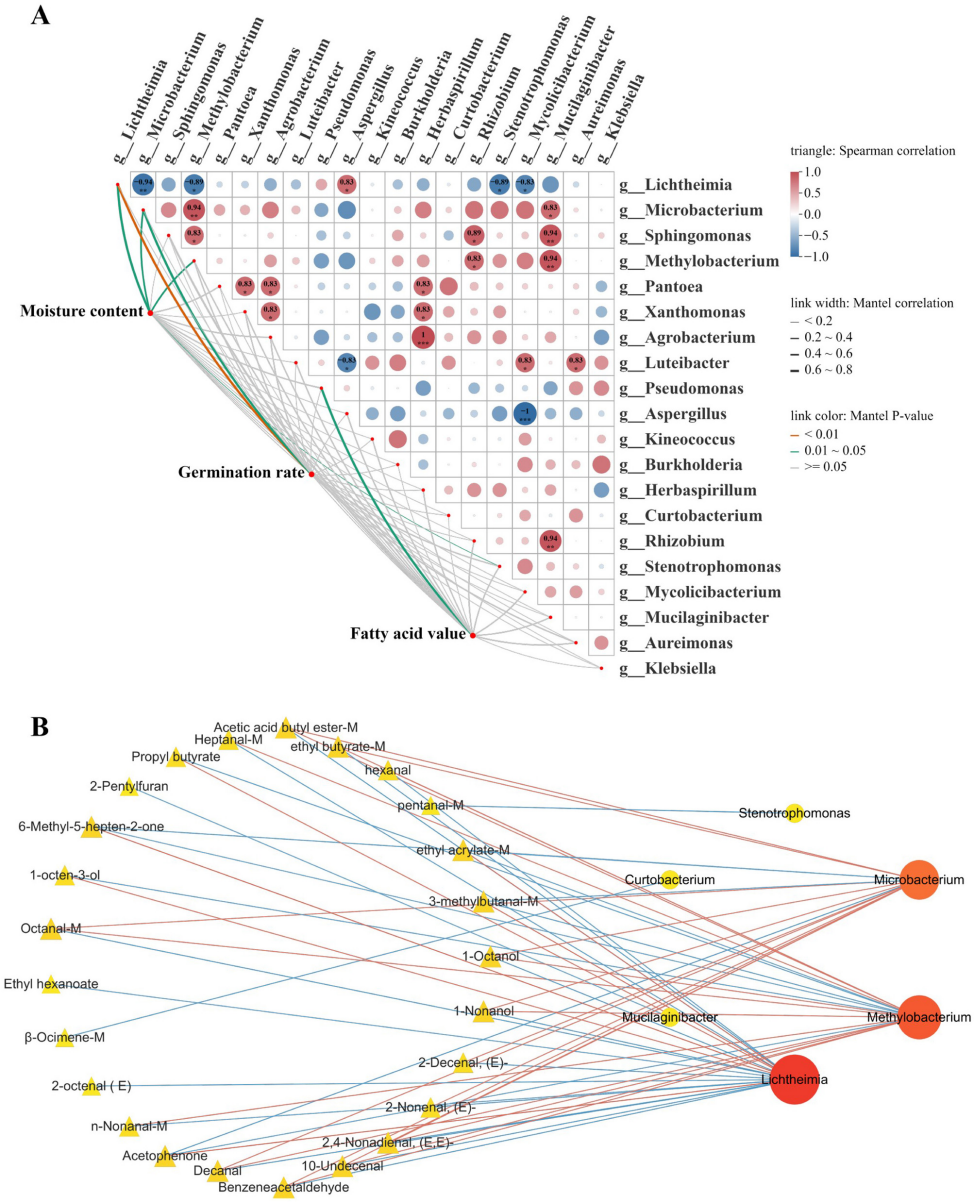
Correlation analysis between microbial communities and physicochemical properties of rice grains during transportation. **(A)** Correlation heatmap between microbial genera and physicochemical indicators; **(B)** association network between microbial genera and key volatile compounds.

Based on six independent biological replicates, Spearman’s rank correlation analysis was conducted to investigate the relationships between microbial genera and physicochemical indicators (Figure 7A). Moisture content was found to be significantly correlated with hydrophilic or highly adaptable genera, including *Lichtheimia*, *Microbacterium*, *Methylobacterium*, and *Stenotrophomonas*. Among them, a significant negative correlation was also observed between *Lichtheimia* and *Microbacterium* and germination rate, suggesting that high-moisture conditions may promote the colonization and proliferation of these taxa. Specifically, the hyphal invasion of *Lichtheimia* might compromise the structural integrity of the seed coat and embryo, thereby hindering germination initiation (34). Similarly, *Microbacterium* likely contributes to the decline in germination rate by secreting organic acids and extracellular enzymes that degrade stored starch and proteins in the endosperm (35).

Notably, a significant positive correlation was exhibited between fatty acid value and the relative abundance of *Pseudomonas*. This relationship may be attributed to a dual mechanism: on one hand, *Pseudomonas* is capable of secreting heat-stable extracellular lipases that catalyze triglyceride hydrolysis (36); on the other hand, free fatty acids accumulated via spontaneous hydrolysis under high-temperature and high-humidity conditions might construct a specific nutritional niche, thereby selecting for and enriching *Pseudomonas* populations that tolerate or prefer such substrates. Furthermore, distinct structural differentiation within the microbial community was identified, with *Lichtheimia* displaying a significant negative correlation with *Microbacterium*. This implies that *Lichtheimia*, as the emerging dominant fungal genus after 12 days of transportation, may have exerted competitive or antagonistic effects on the originally dominant bacteria, leading to a significant restructuring of the microbial community.

### 3.5 Correlation analysis between microbial genera and volatile compounds

Correlation analysis was performed between dominant microbial genera and key volatile compounds (OAV or ROAV > 1), using | R| ≥ 0.70 and *P* < 0.05 as screening thresholds. The results indicated a tight coupling relationship between the microbial community and volatile metabolites during rice transportation, suggesting that microbial-mediated lipid degradation, oxidative reactions, and secondary metabolic processes were key drivers of volatile compound evolution (Figure 7B). For instance, *Pseudomonas*, which was strongly correlated with fatty acid values, likely accelerates lipid hydrolysis by secreting lipases; this process releases free fatty acids as oxidation precursors, thereby contributing to the development of rancid odors (37).

*Microbacterium* showed a close relationship with volatile products derived from lipid oxidation (38), specifically aldehydes and esters such as decanal, (E)-2-nonenal, and acetic acid butyl ester, which predominantly originate from the oxidation or esterification of unsaturated fatty acids (e.g., linoleic and oleic acids) (39). In contrast, *Microbacterium* was negatively correlated with compounds like acetophenone and 3-methylbutanal, indicating distinct metabolic associations with different types of volatile compounds. *Methylobacterium* was also significantly correlated with multiple aldehydes and esters, including hexanal, nonanal, and ethyl butyrate. Considering that esters are typically formed through reactions between fatty acids and alcohols, these associations imply a potential role of *Methylobacterium* in alcohol metabolism and esterification processes, thus influencing the aroma of rice during transportation (40). The fungal genus *Lichtheimia* exhibited a distinct “divergent correlation pattern” with metabolic products. On one hand, it was positively correlated with typical fungal metabolites such as 1-octen-3-ol and 6-methyl-5- hepten-2-one, among which the accumulation of 1-octen-3-ol is widely recognized as an indicator of rice mold development (41). On the other hand, *Lichtheimia* exhibited strong negative correlations with freshness- related aroma compounds, including acetic acid butyl ester and decanal. This dual behavior underscores its crucial role as a key biological indicator of aroma deterioration and mold development in rice. Collectively, *Microbacterium*, *Methylobacterium*, and *Lichtheimia* synergistically contribute to the transition of rice aroma from a fresh profile to deteriorated and mold-associated characteristics during transportation through their specific metabolic associations with various volatile compounds.

## 4 Conclusion

High-temperature and high-humidity transportation accelerates rice quality deterioration through a synergistic mechanism involving moisture absorption, lipid degradation, and microbial succession. Although this study was conducted under laboratory-simulated conditions using a single indica rice cultivar, the results clearly demonstrate a distinct transition from initial physicochemical deterioration–characterized by rising moisture and fatty acid values alongside declining germination rates–to severe degradation dominated by microbial activity. Specifically, the microbial community shifts from a bacteria-dominated ecosystem to one dominated by mold-associated fungi, particularly *Lichtheimia*. This succession, coupled with *Pseudomonas*-mediated lipid hydrolysis, significantly reshapes the volatile profile: fresh lipid-derived aldehydes decrease, while fermentation-related alcohols and esters accumulate. Based on these dynamic changes, seven volatile compounds–3-methylbutanal, pentanal, 1-octen-3-ol, isopentyl alcohol, 2-methyl-1-propanol, ethyl acrylate, and ethyl acetate–were identified as characteristic markers indicative of deterioration. These findings offer a theoretical foundation for quality control and risk management, though future validation across multiple cultivars and real-world scenarios is warranted to confirm the generalizability of these early warning indicators.

## Data Availability

The raw sequencing data supporting the conclusions of this article have been deposited in the NCBI Sequence Read Archive (SRA) under the BioProject accession number PRJNA1425942 and are publicly available at: https://www.ncbi.nlm.nih.gov/bioproject/PRJNA1425942. All other relevant data are available from the corresponding author upon reasonable request.

## Footnotes

1
https://cloud.metware.cn/
