# Supplementary material for: Impacts of high-temperature and humidity transportation on rice quality: an integrated analysis of microbial community succession and flavor compound alterations
Source: Front Nutr. 2026 Mar 5;13:1792369. doi: 10.3389/fnut.2026.1792369 (PMC12999384; doi:10.3389/fnut.2026.1792369)
Supplement: Supplementary file 1 [file Table_1.docx]

Supplementary Material

| No. | Compound | Odor threshold  (μg/kg) | Concentration (μg/kg) | | OAV | |
| --- | --- | --- | --- | --- | --- | --- |
|  |  |  | Control | Treated | Control | Treated |
| Alcohols | | | | | | |
| 1 | 1-Octanol | 22 | 142.24±7.54^a^ | ND | 6.47 | - |
| 2 | 1-Hexanol, 2-ethyl- | 1280 | ND | 186.62±8.46^a^ | - | 0.15 |
| 3 | 2-Tridecanol | - | 26.09±8.77^a^ | ND | - | - |
| 4 | 1-Nonanol | 5.3 | 148.69±6.63^a^ | 15.04±2.52^b^ | 28.05 | 2.84 |
| Aldehydes | | | | | | |
| 1 | 2-Decenal, (E)- | 5 | 76.81±5.79^a^ | ND | 15.36 | - |
| 2 | 2-Nonenal, (E)- | 0.08 | 40.33±4.90^a^ | ND | 504.13 | - |
| 3 | 2,4-Nonadienal, (E,E)- | 0.16 | 33.86±2.99^a^ | ND | 211.63 | - |
| 4 | 10-Undecenal | 3.5 | 55.01±4.18^a^ | ND | 15.72 | - |
| 5 | Benzeneacetaldehyde | 6.3 | 14.75±1.00^a^ | ND | 2.34 | - |
| 6 | Decanal | 0.1 | 96.00±1.69^a^ | 21.14±1.45^b^ | 960.00 | 211.40 |
| Ketones | | | | | | |
| 1 | Acetophenone | 5.629 | ND | 46.49±9.40^a^ | - | 8.26 |
| 2 | 5,9-Undecadien-2-one, 6,10-  dimethyl-, (E)- | 60 | ND | 19.32±3.80^a^ | - | 0.32 |
| 3 | 2-Undecanone, 6,10-dimethyl- | - | 101.85±8.69^a^ | ND | - | - |
| Esters | | | | | | |
| 1 | Heptadecanoic acid, ethyl ester | - | ND | 9.75±1.79^a^ | - | - |
| 2 | Benzoic acid, ethyl ester | 300000 | ND | 192.05±12.38^a^ | - | 0.00 |
| 3 | Benzeneacetic acid, ethyl ester | 100 | 20.94±2.56^b^ | 72.24±6.17^a^ | 0.21 | 0.72 |
| 4 | Octadecanoic acid, ethyl ester | 6440 | 7.21±1.29^a^ | 14.33±9.96^a^ | 0.00 | 0.00 |
| 5 | Pentadecanoic acid, ethyl ester | - | 41.69±4.58^b^ | 67.51±10.56^a^ | - | - |
| 6 | Ethyl 13-methyl-tetradecanoate | - | 16.64±1.55^b^ | 23.71±1.98^a^ | - | - |
| 7 | Nonanoic acid, 9-oxo-,  ethyl ester | - | 20.49±3.37^a^ | 15.92±1.77^a^ | - | - |
| 8 | Ethyl tridecanoate | - | 25.65±1.90^a^ | 19.42±0.17^b^ | - | - |
| 9 | Undecanoic acid, ethyl ester | 1000 | 68.12±5.58^a^ | 31.32±1.13^b^ | 0.07 | 0.03 |
| Alkanes | | | | | | |
| 1 | Pentadecane, 3-methyl- | - | ND | 23.30±2.46^a^ | - | - |
| 2 | Pentadecane, 2,6,10-trimethyl- | - | ND | 11.93±0.78^a^ | - | - |
| 3 | Dodecane | 10000 | ND | 46.27±5.18^a^ | - | 0.00 |
| 4 | Heptadecane | - | 15.93±0.58^a^ | 16.07±1.63^a^ | - | - |
| 5 | 2,6,10-Trimethyltridecane | - | 41.79±3.90^a^ | 28.50±3.54^b^ | - | - |
| 6 | Tridecane, 3-methyl- | - | 61.58±6.86^a^ | 39.25±2.39^b^ | - | - |
| 7 | Tetradecane | 10000 | 101.84±9.27^a^ | 63.75±3.61^b^ | 0.01 | 0.01 |
| 8 | Pentadecane | 13000000 | 55.02±3.50^a^ | 34.02±2.51^b^ | 0.00 | 0.00 |
| 9 | Tridecane, 5-methyl- | - | 15.45±5.28^a^ | 7.00±1.56^a^ | - | - |
| 10 | Hexadecane | 500 | 57.07±3.10^a^ | 22.59±0.54^b^ | 0.11 | 0.05 |
| 11 | Undecane, 3-methyl- | - | 23.93±2.99^a^ | 6.69±0.89^b^ | - | - |

Table S1. Volatile compounds identified in rice grains before and after transportation by GC-MS/MS, including odor thresholds and OAV.

**Note:** Different lowercase letters in the same row indicate significant differences between groups (*P* < 0.05). ND indicates not detected.

Table S2. Volatile compounds identified in rice grains before and after transportation by GC-IMS, including odor thresholds and ROAV.

| No. | Compound | Odor threshold  (μg/L) | Relative content（%） | | ROAV | |
| --- | --- | --- | --- | --- | --- | --- |
|  |  |  | Control | Treated | Control | Treated |
| Alcohols | | | | | | |
| 1 | 1-octen-3-ol | 0.01 | 1.22±0.04^b^ | 1.95±0.17^a^ | 1.438 | 5.175 |
| 2 | n-Hexanol | 0.5 | 3.54±0.11^a^ | 3.88±0.33^a^ | 0.083 | 0.206 |
| 3 | 1-pentanol | 4 | 5.82±0.12^a^ | 3.22±0.01^b^ | 0.017 | 0.021 |
| 4 | 2,3-Butanediol | 100 | 1.09±0.01^b^ | 2.46±0.06^a^ | 0.000 | 0.001 |
| 5 | Isopentyl alcohol | ND | 3.54±0.08^b^ | 8.41±0.42^a^ | - | - |
| 6 | 2-methyl-1-propanol | 6.5 | 1.76±0.03^b^ | 3.69±0.27^a^ | 0.003 | 0.015 |
| 7 | 1-propanol | 9 | 1.81±0.13^b^ | 2.70±0.17^a^ | 0.002 | 0.008 |
| 8 | 1-butanol | 4.3 | 1.93±0.06^b^ | 3.89±0.07^a^ | 0.005 | 0.024 |
| Aldehydes | | | | | | |
| 1 | n-Nonanal | 0.001 | 5.76±0.36^a^ | 3.77±0.36^b^ | 67.849 | 100 |
| 2 | 2-octenal ( E) | 0.003 | 0.73±0.03^a^ | 0.44±0.02^b^ | 2.862 | 3.886 |
| 3 | Octanal | 0.0006 | 3.69±0.03^a^ | 1.42±0.10^b^ | 72.355 | 62.736 |
| 4 | 2-heptenal ( E) | 0.04 | 1.14±0.06^a^ | 1.07±0.07^a^ | 0.335 | 0.713 |
| 5 | Heptanal | 0.002 | 2.97±0.01^a^ | 1.30±0.13^b^ | 17.495 | 17.274 |
| 6 | 2-Hexenal | 0.03 | 0.53±0.02^a^ | 0.41±0.01^b^ | 0.21 | 0.366 |
| 7 | hexanal | 0.02 | 1.11±0.03^a^ | 0.82±0.06^b^ | 0.655 | 1.084 |
| 8 | pentanal | 0.012 | 2.57±0.14^a^ | 2.69±0.04^a^ | 2.519 | 5.948 |
| 9 | 3-methylbutanal | 0.0011 | 2.54±0.14^b^ | 3.45±0.07^a^ | 27.212 | 83.248 |
| Ketones | | | | | | |
| 1 | 6-Methyl-5-hepten-2-one | 0.068 | 2.07±0.15^b^ | 2.94±0.16^a^ | 0.358 | 1.146 |
| 2 | Cyclohexanone | 20 | 1.49±0.08^b^ | 1.77±0.02^a^ | 0.001 | 0.002 |
| 3 | 2-Propanone | 300 | 3.68±0.05^b^ | 3.19±0.17^a^ | 0.000 | 0.000 |
| Esters | | | | | | |
| 1 | Butanoic acid, 3-methylbutyl ester | ND | 8.22±0.88^a^ | 3.12±0.25^b^ | - | - |
| 2 | isoamyl butyrate | 0.5 | 2.26±0.18^b^ | 3.42±0.30^a^ | 0.053 | 0.181 |
| 3 | Ethyl hexanoate | 0.001 | 1.12±0.05^a^ | 0.87±0.04^b^ | 13.175 | 23.161 |
| 4 | ethyl 2-methylpentanoate | 0.05 | 1.09±0.05^b^ | 1.74±0.04^a^ | 0.257 | 0.924 |
| 5 | 2-Methylbutanol acetate | ND | 3.61±0.14^a^ | 2.35±0.17^b^ | - | - |
| 6 | Propyl butyrate | 0.018 | 1.13±0.02^b^ | 1.36±0.06^a^ | 0.737 | 2.004 |
| 7 | Acetic acid butyl ester | 0.006 | 13.89±0.16^a^ | 4.56±0.48^b^ | 27.26 | 20.163 |
| 8 | ethyl butyrate | 0.001 | 8.49±0.17^a^ | 3.17±0.03^b^ | 100 | 84.037 |
| 9 | Butyl formate | 0.8 | 0.37±0.01^b^ | 1.74±0.12^a^ | 0.005 | 0.058 |
| 10 | ethyl acetate | 5 | 3.44±0.08^b^ | 8.73±0.23^a^ | 0.008 | 0.046 |
| 11 | n-Propyl acetate | 2 | 0.64±0.02^a^ | 0.23±0.01^b^ | 0.004 | 0.003 |
| 12 | ethyl acrylate | 0.005 | 4.12±0.09^b^ | 11.94±0.46^a^ | 9.701 | 63.324 |
| Others | | | | | | |
| 1 | β-Ocimene | 0.015 | 1.83±1.08^a^ | 2.45±0.39^a^ | 1.443 | 4.332 |
| 2 | 2-Pentylfuran | 0.006 | 0.81±0.01^a^ | 0.85±0.04^a^ | 1.586 | 3.778 |
